# Antibiotic-Prescribing Practices for Management of Childhood Diarrhea in 3 Sub-Saharan African Countries: Findings From the Vaccine Impact on Diarrhea in Africa (VIDA) Study, 2015–2018

**DOI:** 10.1093/cid/ciac980

**Published:** 2023-04-19

**Authors:** Alex O Awuor, Billy Ogwel, Helen Powell, Jennifer R Verani, Samba O Sow, M Jahangir Hossain, John B Ochieng, Jane Juma, Leslie P Jamka, Anna Roose, Sanogo Doh, Emily L Deichsel, Uma Onwuchekwa, Adama Mamby Keita, Martin Antonio, Joquina Chiquita M Jones, Syed M A Zaman, Henry Badji, Irene N Kasumba, Dilruba Nasrin, James A Platts-Mills, Eric R Houpt, David M Berendes, Ciara E Sugerman, Marc-Alain Widdowson, Sharon M Tennant, Eric D Mintz, Richard Omore, Karen L Kotloff

**Affiliations:** Kenya Medical Research Institute, Center for Global Health Research (KEMRI-CGHR), Kisumu, Kenya; Kenya Medical Research Institute, Center for Global Health Research (KEMRI-CGHR), Kisumu, Kenya; Center for Vaccine Development and Global Health, University of Maryland School of Medicine, Baltimore, Maryland, USA; Department of Pediatrics, University of Maryland School of Medicine, Baltimore, Maryland, USA; Division of Global Health Protection, Centers for Disease Control and Prevention, Nairobi, Kenya; Centre pour le Développement des Vaccins du Mali (CVD-Mali), Bamako, Mali; Medical Research Council Unit–The Gambia at the London School of Hygiene & Tropical Medicine, Banjul, The Gambia; Kenya Medical Research Institute, Center for Global Health Research (KEMRI-CGHR), Kisumu, Kenya; Kenya Medical Research Institute, Center for Global Health Research (KEMRI-CGHR), Kisumu, Kenya; Center for Vaccine Development and Global Health, University of Maryland School of Medicine, Baltimore, Maryland, USA; Department of Medicine, University of Maryland School of Medicine, Baltimore, Maryland, USA; Center for Vaccine Development and Global Health, University of Maryland School of Medicine, Baltimore, Maryland, USA; Department of Pediatrics, University of Maryland School of Medicine, Baltimore, Maryland, USA; Centre pour le Développement des Vaccins du Mali (CVD-Mali), Bamako, Mali; Center for Vaccine Development and Global Health, University of Maryland School of Medicine, Baltimore, Maryland, USA; Department of Pediatrics, University of Maryland School of Medicine, Baltimore, Maryland, USA; Centre pour le Développement des Vaccins du Mali (CVD-Mali), Bamako, Mali; Centre pour le Développement des Vaccins du Mali (CVD-Mali), Bamako, Mali; Medical Research Council Unit–The Gambia at the London School of Hygiene & Tropical Medicine, Banjul, The Gambia; Medical Research Council Unit–The Gambia at the London School of Hygiene & Tropical Medicine, Banjul, The Gambia; Medical Research Council Unit–The Gambia at the London School of Hygiene & Tropical Medicine, Banjul, The Gambia; Medical Research Council Unit–The Gambia at the London School of Hygiene & Tropical Medicine, Banjul, The Gambia; Center for Vaccine Development and Global Health, University of Maryland School of Medicine, Baltimore, Maryland, USA; Department of Medicine, University of Maryland School of Medicine, Baltimore, Maryland, USA; Center for Vaccine Development and Global Health, University of Maryland School of Medicine, Baltimore, Maryland, USA; Department of Medicine, University of Maryland School of Medicine, Baltimore, Maryland, USA; Division of Infectious Diseases and International Health, Department of Medicine, University of Virginia, Charlottesville, Virginia, USA; Division of Infectious Diseases and International Health, Department of Medicine, University of Virginia, Charlottesville, Virginia, USA; Division of Foodborne, Waterborne, and Environmental Diseases, Centers for Disease Control and Prevention, Atlanta, Georgia, USA; Division of Foodborne, Waterborne, and Environmental Diseases, Centers for Disease Control and Prevention, Atlanta, Georgia, USA; Division of Global Health Protection, Centers for Disease Control and Prevention, Nairobi, Kenya; Center for Vaccine Development and Global Health, University of Maryland School of Medicine, Baltimore, Maryland, USA; Department of Medicine, University of Maryland School of Medicine, Baltimore, Maryland, USA; Division of Foodborne, Waterborne, and Environmental Diseases, Centers for Disease Control and Prevention, Atlanta, Georgia, USA; Kenya Medical Research Institute, Center for Global Health Research (KEMRI-CGHR), Kisumu, Kenya; Center for Vaccine Development and Global Health, University of Maryland School of Medicine, Baltimore, Maryland, USA; Department of Pediatrics, University of Maryland School of Medicine, Baltimore, Maryland, USA; Department of Medicine, University of Maryland School of Medicine, Baltimore, Maryland, USA

**Keywords:** diarrhea, antibiotic, prescription, children, Africa

## Abstract

**Background:**

Despite antibiotic prescription being recommended for dysentery and suspected cholera only, diarrhea still triggers unwarranted antibiotic prescription. We evaluated antibiotic-prescribing practices and their predictors among children aged 2–59 months in the Vaccine Impact on Diarrhea in Africa (VIDA) Study performed in The Gambia, Mali, and Kenya.

**Methods:**

VIDA was a prospective case-control study (May 2015–July 2018) among children presenting for care with moderate-to-severe diarrhea (MSD). We defined inappropriate antibiotic use as prescription or use of antibiotics when not indicated by World Health Organization (WHO) guidelines. We used logistic regression to assess factors associated with antibiotic prescription for MSD cases who had no indication for an antibiotic, at each site.

**Results:**

VIDA enrolled 4840 cases. Among 1757 (36.3%) who had no apparent indication for antibiotic treatment, 1358 (77.3%) were prescribed antibiotics. In The Gambia, children who presented with a cough (adjusted odds ratio [aOR]: 2.05; 95% confidence interval [95% CI]: 1.21–3.48) were more likely to be prescribed an antibiotic. In Mali, those who presented with dry mouth (aOR: 3.16; 95% CI: 1.02–9.73) were more likely to be prescribed antibiotics. In Kenya, those who presented with a cough (aOR: 2.18; 95% CI: 1.01–4.70), decreased skin turgor (aOR: 2.06; 95% CI: 1.02–4.16), and were very thirsty (aOR: 4.15; 95% CI: 1.78–9.68) were more likely to be prescribed antibiotics.

**Conclusions:**

Antibiotic prescription was associated with signs and symptoms inconsistent with WHO guidelines, suggesting the need for antibiotic stewardship and clinician awareness of diarrhea case-management recommendations in these settings.

## Teaser

Inappropriate antibiotic prescribing for diarrheal diseases can promote antimicrobial resistance. In VIDA, unnecessary or incorrect antibiotics were prescribed for 77% of diarrhea and 41% of dysentery cases. Interventions are needed to promote judicious adherence to international guidelines in low-/middle-income countries.

The overuse or misuse of antibiotics when deemed unnecessary or not recommended by the international and national treatment guidelines is a major global public health threat [1]. Diarrheal diseases remain a common clinical syndrome in which inappropriate antibiotics are used, even though most of these illnesses are caused by viruses or self-limited bacterial infections. Accordingly, the World Health Organization (WHO) Integrated Management of Childhood Infections (IMCI) handbook does not recommend antibiotics for the majority of diarrheal episodes, with the exception of bloody diarrhea (with the aim of treating shigellosis, the most common cause), suspected cholera with severe dehydration, or co-existing severe acute malnutrition (SAM) [2]. Inappropriate use of antibiotics for diarrheal diseases or other conditions can promote antimicrobial resistance (AMR) to both diarrheal and nondiarrheal pathogens [3], increase healthcare costs to both providers and families [4], and increase morbidity and risks of adverse reactions, including prolonged hospitalization [5–9]. Antibiotic-resistant strains of *Shigella*, for example, have emerged, leaving few options for effective, affordable therapy [10–12].

Although AMR patterns can differ by geographic region [13], resistant strains have spread globally [14] and exert the greatest impact in low-income countries [15, 16]. Resistance to essential antibiotics continues to increase in sub-Saharan Africa [17]—a setting where knowledge of factors driving antibiotic prescription remains poorly understood. This was exemplified by a study of AMR among atypical enteropathogenic *Escherichia coli* isolated from stool samples from children younger than 5 years old with and without diarrhea in 7 developing countries in sub-Saharan Africa and South Asia participating in the Global Enteric Multicenter Study (GEMS) [18]. Using a combination of phenotyping and genomics, these investigators found that 65% of isolates displayed resistance to 3 or more drug classes over a 3-year period. To better understand the drivers of antibiotic prescribing for diarrhea in sub-Saharan Africa, we examined findings from the Vaccine Impact on Diarrhea in Africa (VIDA) Study, a 3-year follow-on study to GEMS that prospectively collected data on the clinical management of MSD among children younger than 5 years of age attending healthcare facilities at 3 sites in sub-Saharan Africa [19].

## METHODS

### Study Design

The aim of this analysis was to assess the prevalence of inappropriate antibiotic-prescribing practices and their predictors among cases with MSD aged 2–59 months nested within VIDA, a case-control study designed to elucidate the incidence, etiology, and adverse clinical consequences of MSD in children aged 0–59 months residing in censused populations in Basse and Bansang, The Gambia; Bamako, Mali; and Siaya County, Kenya following rotavirus vaccine introduction. VIDA used comparable methods to GEMS [20]. The study methods are described in detail previously [21] and more recently [20].

### Case Definition and Recruitment Methods

In brief, during a 36-month period from May 2015 to July 2018, each site enrolled children 0–59 months of age belonging to a censused population when they sought care at local Sentinel Health Centers (SHCs). This analysis included a subset of these children in 3 age strata (2–11 months, 12–23 months, and 24–59 months) with an acute, new episode of MSD, defined as 3 abnormally loose or watery stools in the previous 24 hours, accompanied by at least 1 of the following: sunken eyes, skin tenting, intravenous rehydration, or hospitalization. To study antibiotic use in those children with MSD for whom antibiotics were not recommended, children enrolled in the main VIDA study were excluded from this analysis if they were younger than 2 months of age (because WHO recommendations do not address this age group [2]) or who had a WHO indication for antibiotics based on clinician diagnosis, including dysentery, pneumonia, cholera, meningitis, or other invasive bacterial infections; otitis media, tonsillitis, or pharyngitis; or SAM [2]. The list of the definitions for the exclusion criteria are shown in Supplementary Table 1. Note that, per IMCI, cough or difficulty breathing in the absence of chest in-drawing or tachypnea was not considered an indication for antibiotics.

### Data Collection, Study Definitions, and Statistical Analysis Methods

Information on demographic, epidemiological, and clinical characteristics prior to presentation at the SHC was collected from the child's primary caretaker at enrollment on standardized forms. We recorded the study clinician's assessment of the child at enrollment and the medical management during the child's stay at the SHC, including antibiotic prescription data. Antibiotic prescription was defined as either having been given an antibiotic at the facility or receiving a prescription for home use. We defined probable unnecessary antibiotic use as prescription or use of antibiotics when not indicated by WHO guidelines. Undernutrition was defined as children who were wasted/very thin based on clinician judgement but who did not meet criteria for SAM. Clinical fever was defined as a temperature 37.5**°**C or higher. Tachypnea was defined as respiratory rate in breaths/minute greater than 50 in infants aged 2–12 months and greater than 40 in children aged 1–5 years.

Using either chi-square or Fisher`s exact test, as appropriate, we compared proportions of MSD cases with various clinical and demographic characteristics among those who were or were not given a prescription for antibiotics. Medians for continuous variables were compared using a Wilcoxon rank-sum test. We performed logistic regression with antibiotic prescription as the outcome and all demographic and clinical symptoms as potential covariates separately for each site to allow for site heterogeneity (clinical presentation, demographic, and national guidelines). For all analyses, a *P* value less than .05 was considered statistically significant. Data analysis was performed using Stata/SE 16.0 (StataCorp, College Station, TX, USA).

### Ethical Approval

This study was approved by the ethical review committees at the University of Maryland, Baltimore (HP-00062472); the Centers for Disease Control and Prevention (CDC) (reliance agreement 6729); The Gambia Government/Medical Research Council/Gambia at the London School of Hygiene and Tropical Medicine (1409); the Comité d'Ethique de la Faculté de Médecine, de Pharmacie, et d'Odonto-Stomatologie, Bamako, Mali (no number); and the Kenya Medical Research Institute Scientific and Ethics Review Unit in Siaya County, Kenya (SSE 2996). Written informed consent was obtained from the parent(s) or primary caretaker(s) of each child who met eligibility criteria before any research activities were performed.

## RESULTS

### Participants

VIDA enrolled a total of 4840 MSD cases at the 3 study sites combined. The number of cases who met exclusion criteria for this analysis was 806 of 1678 (48.0%) in The Gambia, 468 of 1608 (29.1%) in Mali, and 1066 of 1554 (68.6%) in Kenya (Supplementary Table 2). The leading causes of exclusion across the sites were severe pneumonia and severe febrile disease.

### Antibiotic Prescription Patterns

**Figure 1.**
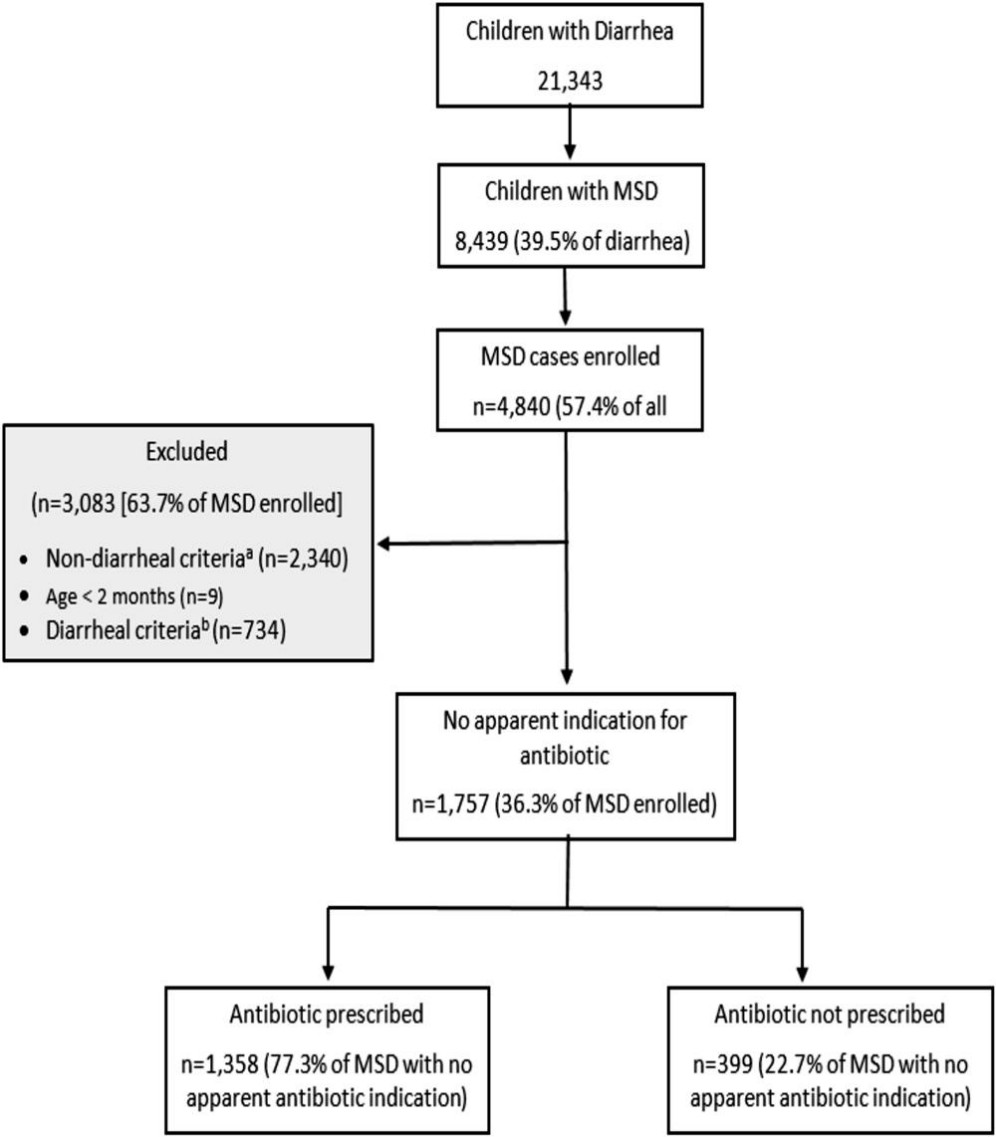
Enrollment and antibiotic prescription flowchart in VIDA: 2015–2018. Abbreviations: IMCI, Integrated Management of Childhood Infections; MSD, moderate-to-severe diarrhea; SAM, severe acute malnutrition; VIDA, Vaccine Impact on Diarrhea in Africa. ^a^Nondiarrheal criteria for exclusion: (1) clinical diagnosis—pneumonia, meningitis, bacterial infection, sepsis, pharyngitis, otitis media, skin infection, impetigo; (2) IMCI criterial for antibiotics—uncomplicated SAM, pneumonia, SAM with complications, very severe febrile disease. ^b^Diarrheal criteria for exclusion: dysentery, cholera.

Among the MSD cases enrolled, 734 of 4840 (15.2%) had a diarrheal indication for antibiotic prescription and, of these, 732 of 734 (99.7%) had dysentery. Among those with dysentery, 641 of 732 (87.6%) were prescribed antibiotics. The most commonly prescribed antibiotics among dysenteric cases were ciprofloxacin (365/428 [85.3%]) and metronidazole (27/428 [6.3%]) in The Gambia, metronidazole (50/60 [83.3%]) and cotrimoxazole (41/60 [68.3%]) in Mali, and ciprofloxacin (67/153 [43.8%]) and metronidazole (50/153 [32.7%]) in Kenya. A total of 1757 (36.3%) enrolled MSD cases had no clinical indication for antibiotics according to our study definition and consistent with IMCI guidelines. Of those who had no apparent indication for antibiotics, 1358 of 1757 (77.3%) were prescribed antibiotics (Figure 1).

The highest proportion of MSD cases with no apparent antibiotic indication who were prescribed antibiotics was observed in Mali (1055/1075 [98.1%]), followed by The Gambia (232/403 [57.6%]) and Kenya (70/279 [25.1%]) (Table 1). The most commonly prescribed antibiotics were cotrimoxazole (880/1055 [83.4%]) and metronidazole (810/1055 [76.8%]) in Mali, ciprofloxacin (109/232 [47.0%]) and metronidazole (68/232 [29.3%]) in The Gambia, and cotrimoxazole (29/70 [41.4%]) and metronidazole (19/70 [27.1%]) in Kenya.

**Table 1.** Antibiotics Prescribed for Moderate-to-Severe Diarrhea Cases Without an Apparent Indication for Antibiotic Treatment Stratified by Site in VIDA, 2015–2018

	The Gambia	Mali	Kenya
No. without apparent indication for antibiotics	403	1075	279
No. (%) prescribed antibiotics without apparent indication	232 (57.6)	1056 (98.2)	70 (25.1)
No. (%) of MSD cases prescribed, by antibiotic, among those without an apparent indication			
ȃCiprofloxacin	109 (47)	1 (0)	1 (1)
ȃMetronidazole	68 (29)	810 (77)	19 (27)
ȃCotrimoxazole	41 (18)	880 (83)	29 (41)
ȃChloramphenicol	21 (9)	0 (0)	0 (0)
ȃAzithromycin	10 (4)	7 (1)	0 (0)
ȃErythromycin	6 (3)	22 (2)	12 (17)
ȃAmoxycillin	4 (2)	8 (1)	9 (13)
ȃAmpicillin	4 (2)	0 (0)	0 (0)
ȃGentamycin	3 (1)	8 (1)	0 (0)
ȃPenicillin	1 (0)	0 (0)	0 (0)
ȃNalidixic acid	1 (0)	0 (0)	0 (0)
ȃCeftriaxone	0 (0)	49 (5)	0 (0)
ȃCephalosporin	0 (0)	12 (1)	0 (0)
ȃPivmecillinam	0 (0)	0 (0)	0 (0)
ȃOther	0 (0)	0 (0)	1 (1)^a^

Abbreviations: MSD, moderate-to-severe diarrhea; VIDA, Vaccine Impact on Diarrhea in Africa.

Tetracycline.

The distribution of characteristics of MSD cases with no antibiotic indication who did and did not receive antibiotic prescription stratified by site is shown in Table 2. This univariate analysis showed that children with cough were significantly more likely to receive a prescription for inappropriate antibiotics in The Gambia (28.9% vs 19.9%; *P* = .039). A similar pattern was seen in Kenya for cough (35.7% vs 23.9%; *P* = .054) and difficulty breathing (5.7% vs 2.4%; *P* = .048). Children with signs of dehydration were less likely to receive an inappropriate antibiotic prescription in The Gambia (very thirsty: 78.0% vs 86.0% [*P* = .043]; dry mouth: 65.9% vs 76.6% [*P* = .02]) but more likely in Mali (dry mouth: 66.1% vs 42.1%; *P* = .029) and Kenya (very thirsty: 85.7% vs 62.2% [*P* < .0001]; decreased skin turgor: 50.0% vs 34.9% [*P* = .025]). In The Gambia, younger children appeared more likely to receive an inappropriate antibiotic prescription. In Kenya, children with fever (14.3% vs 6.2%; *P* = .024) were more likely to receive an inappropriate antibiotic prescription, whereas in The Gambia, children with belly pain were less likely (38.8% vs 52.0%; *P* = .004).

**Table 2.** Characteristics of Moderate-to-Severe Diarrhea Cases Without an Indication for Antibiotic Prescription Stratified by Site in VIDA, 2015–2018

Characteristics	The Gambia			Mali			Kenya		
	Antibiotic Prescribed (n = 232)	Antibiotic Not Prescribed (n = 171)	*P*	Antibiotic Prescribed (n = 1056)	Antibiotic Not Prescribed (n = 19)	*P*	Antibiotic Prescribed (n = 70)	Antibiotic Not Prescribed (n = 209)	*P*
**Demographic features**									
ȃAge									
ȃȃ2–11 months	84 (36.2)	41 (24)	.022*	364 (34.5)	5 (26.3)	.754	24 (34.3)	66 (31.6)	.764
ȃȃ12–23 months	73 (31.5)	71 (41.5)		357 (33.8)	7 (36.8)		25 (35.7)	85 (40.7)	
ȃȃ24–59 months	75 (32.3)	59 (34.5)		335 (31.7)	7 (36.8)		21 (30)	84 (36.2)	
ȃMedian [IQR] age, years	16 [10–25]	19 [12–28]	.112	16 [10–26]	22 [11–33]	.451	15.5 [10–25]	16 [9–24]	.656
ȃMale	116 (50)	88 (51.5)	.772	531 (50.3)	14 (73.7)	.062	35 (50)	108 (51.7)	.808
**Clinical features**									
ȃBy history									
ȃȃBelly pain	90 (38.8)	89 (52)	.004*	261 (24.7)	2 (10.5)	.263	40 (57.1)	102 (48.8)	.356
ȃȃCough	67 (28.9)	34 (19.9)	.039*	188 (17.8)	5 (26.3)	.362	25 (35.7)	50 (23.9)	.054
ȃȃDifficulty breathing	0 (0)	1 (0.6)	.424	5 (0.5)	0 (0)	1.000	4 (5.7)	3 (1.4)	.048*
ȃȃMaximum loose stools per 24 hours									
ȃȃȃ3	35 (15.1)	26 (15.2)	1.000	470 (44.5)	9 (47.4)	.954	15 (21.4)	45 (21.5)	.002*
ȃȃȃ4–5	169 (72.8)	125 (73.1)		537 (50.9)	9 (47.4)		29 (41.4)	127 (60.8)	
ȃȃȃ≥6	28 (12.1)	20 (11.7)		49 (4.6)	1 (5.3)		26 (37.1)	37 (17.7)	
ȃȃDiarrhea, median [IQR], days	2 [2, 3]	3 [2, 3]	.630	3 [2, 3]	3 [2, 3]	.826	3 [2, 3]	3 [2–4]	.424
ȃȃVomiting	65 (28)	42 (24.6)	.437	106 (10)	3 (15.8)	.430	17 (24.3)	64 (30.6)	.312
ȃAt enrollment									
ȃȃVery thirsty	181 (78)	147 (86)	.043*	1039 (98.4)	19 (100)	1.000	60 (85.7)	130 (62.2)	<.0001*
ȃȃSunken eyes	229 (98.7)	168 (98.2)	.702	1045 (99)	19 (100)	1.000	65 (92.9)	192 (91.9)	.790
ȃȃȃDry mouth									
ȃȃȃȃNormal	79 (34.1)	40 (23.4)	.020*	358 (33.9)	11 (57.9)	.029*	1 (1.4)	1 (0.5)	.440
ȃȃȃȃDry/very dry	153 (65.9)	131 (76.6)		698 (66.1)	8 (42.1)		69 (98.6)	208 (99.5)	
ȃȃȃSkin pinch									
ȃȃȃȃNormal	217 (93.5)	162 (94.7)	.614	744 (70.5)	15 (78.9)	.612	35 (50)	136 (65.1)	.025*
ȃȃȃȃSlow/very slow	15 (6.5)	9 (5.3)		312 (29.5)	4 (21.1)		35 (50)	73 (34.9)	
ȃȃȃMental status									
ȃȃȃȃNormal	216 (93.1)	161 (94.2)	.845	954 (90.3)	17 (89.5)	.469	37 (52.9)	102 (48.8)	.557
ȃȃȃȃIrritable/restless	11 (4.7)	8 (4.7)		78 (7.4)	1 (5.3)		33 (47.1)	107 (51.2)	
ȃȃȃȃLethargic/unconscious	5 (2.2)	2 (1.2)		24 (2.3)	1 (5.3)				
ȃȃȃUndernutrition	17 (7.3)	12 (7)	.905	149 (14.1)	6 (31.6)	.032*	8 (11.4)	16 (7.7)	.330
ȃȃȃTachypnea	15 (6.5)	10 (5.8)	.799	36 (3.4)	0 (0)	1.000	9 (12.9)	16 (7.7)	.226
ȃȃȃFever (>37.5°C)	30 (12.9)	19 (11.1)	.581	109 (10.3)	1 (5.3)	.712	10 (14.3)	13 (6.2)	.034*
ȃȃȃHospitalization	8 (3.4)	3 (1.8)	.367	0 (0)	0 (0)		7 (10)	14 (6.7)	.382

Data are presented as no. (%) unless otherwise indicated. *Significant, *P* < .05. Abbreviations: IQR, interquartile range; VIDA, Vaccine Impact on Diarrhea in Africa.

### Factors Associated With Antibiotic Prescription by Multivariable Analysis

The trends observed in the univariate, bivariate, and multivariable analyses were consistent and generally produced concordant statistically significant associations with some exceptions. For example, in the multivariable analyses, there was no association between antibiotic prescribing and age or fever, whereas children who appeared irritable in Kenya were marginally less likely to receive a prescription.

Cases of MSD with no antibiotic indication in The Gambia who presented with cough in the absence of respiratory distress were more likely than those without cough to have an antibiotic prescribed (adjusted odds ratio [aOR]: 2.05; 95% confidence interval [CI]: 1.21–3.48); those who presented with belly pain (aOR: .53; 95% CI: .35–.81) and dry mouth (aOR: .56; 95% CI: .33–.94) were less likely to have an antibiotic prescribed than those without these symptoms. In Mali, MSD cases who presented with dry mouth were more likely to have an antibiotic prescribed compared with those who did not (aOR: 3.16; 95% CI: 1.02–9.73). In Kenya, a cough (aOR: 2.18; 95% CI: 1.01–4.70), decreased skin turgor (aOR: 2.06; 95% CI: 1.02–4.16), and the child being very thirsty (aOR: 4.15; 95% CI: 1.78–9.68) were positively associated with apparent unnecessary antibiotic prescribing among MSD cases with no antibiotic indication, whereas those who were restless/irritable (aOR: .50; 95% CI: .26–.99) were less likely to have an antibiotic prescribed (Table 3).

**Table 3.** Factors Associated With Antibiotic Prescription by Logistic Regression Among Moderate-to-Severe Diarrhea Cases Without an Indication in VIDA, 2015–2018

Characteristics	The Gambia				Mali				Kenya			
	Bivariate		Multivariable		Bivariate		Multivariable		Bivariate		Multivariable	
	Unadjusted OR (95% CI)	*P*	aOR (95% CI)	*P*	Unadjusted OR (95% CI)	*P*	aOR (95% CI)	*P*	Unadjusted OR (95% CI)	*P*	aOR (95% CI)	*P*
**Demographics**												
ȃAge	.99 (.97–1.00)	.129	.99 (.97–1.01)	.484	.98 (.95–1.02)	.337	.97 (.94–1.01)	.144	1.01 (.98–1.03)	.636	1.00 (.98–1.03)	.772
ȃMale^a^	.94 (.64–1.40)	.772	.90 (.59–1.36)	.611	.36 (.13–1.01)	.052	.33 (.10–1.06)	.062	.93 (.54–1.61)	.808	.82 (.44–1.55)	.549
**Clinical features**												
ȃBy history												
ȃȃBelly pain^b^	.56 (.37–0.83)	.004*	.53 (.35–.81)	.004*	2.52 (.57–11.15)	.224	2.83 (.62–13.0)	.181	1.29 (.75–2.24)	.357	1.33 (.70–2.51)	.384
ȃȃCough^b^	1.64 (1.02–2.62)	.040*	2.05 (1.21–3.48)	.008*	.61 (.22–1.70)	.343	.43 (.13–1.49)	.185	1.77 (.99–3.17)	.056	2.18 (1.01–4.70)	.047*
ȃȃDifficulty breathing^b^	...		...		...		...		4.16 (0.91–19.07)	.066	4.09 (.69–24.06)	.120
ȃȃMaximum loose stools per 24 hours												
ȃȃȃ3	Ref		Ref		Ref		Ref		Ref		Ref	
ȃȃȃ4–5	1.00 (.58–1.75)	.988	.98 (.53–1.79)	.937	1.14 (.45–2.90)	.779	1.48 (.48–4.53)	.497	.69 (.34–1.39)	.296	.76 (.35–1.68)	.499
ȃȃȃ≥6	1.04 (.48–2.24)	.920	1.04 (.44–2.42)	.933	.94 (.12–7.56)	.952	1.07 (.11–10.68)	.953	2.11 (.98–4.55)	.058	1.73 (.71–4.21)	.226
ȃȃDiarrhea, days	1.03 (.84–1.25)	.792	1.07 (.86–1.32)	.559	.83 (.51–1.34)	.434	.83 (.48–1.42)	.488	.92 (.74–1.13)	.418	.86 (.66–1.12)	.278
ȃȃVomiting^b^	1.20 (.76–1.88)	.438	1.38 (.80–2.36)	.246	.60 (.17–2.08)	.415	.22 (.05–1.00)	.050	.73 (.39–1.35)	.313	1.07 (.46–2.51)	.871
ȃAt enrollment												
ȃȃVery thirsty^b^	.58 (.34–.99)	.039*	.66 (.37–1.20)	.176	...		...		4.38 (1.99–9.66)	<.0001*	4.15 (1.78–9.68)	.001*
ȃȃSunken eyes^b^	.89 (.25–3.22)	0.863	2.01 (.37–11.09)	.421	...		...		1.15 (.44–3.04)	.776	2.33 (.71–7.66)	.162
ȃȃȃDry mouth												
ȃȃȃȃNormal	Ref		Ref		Ref		Ref		Ref		Ref	
ȃȃȃȃDry/very dry	.59 (.38–.92)	.021*	.56 (.33–.94)	.028*	2.68 (1.07–6.72)	.036*	3.16 (1.02–9.73)	.045*	.33 (.02–5.37)	.437	...	...
ȃȃȃSkin pinch												
ȃȃȃȃNormal	Ref		Ref		Ref		Ref		Ref		Ref	
ȃȃȃȃSlow/very slow	1.24 (.53–2.91)	.615	1.22 (.48–3.11)	.683	1.57 (.52–4.78)	.424	.78 (.22–2.68)	.688	1.86 (1.08–3.22)	.026*	2.06 (1.02–4.16)	.044*
ȃȃȃMental status												
ȃȃȃȃNormal	Ref		Ref		Ref		Ref		Ref		Ref	
ȃȃȃȃIrritable/restless	1.03 (.40–2.61)	.959	1.13 (.43–2.98)	.809	1.39 (.18–10.58)	.751	...		.85 (.49–1.46)	.557	.50 (.26–.99)	.048*
ȃȃȃȃLethargic/unconscious	1.86 (.36–9.73)	.460	1.62 (.24–10.84)	.619	.43 (.05–3.35)	.418	...		...		...	
ȃȃȃUndernutrition^b^	1.05 (.49–2.26)	.905	1.10 (.45–2.28)	.977	.36 (.13–.95)	.039*	.36 (.12–1.10)	.074	1.56 (.64–3.81)	.333	1.03 (.33–3.19)	.963
ȃȃȃTachypnea^b^	1.11 (.49–2.54)	.800	1.27 (.50–3.25)	.614	...		...		1.78 (.75–4.23)	.192	2.21 (.73–6.68)	.158
ȃȃȃFever (>37.5°C)	1.19 (.64–2.19)	.192	1.06 (.54–2.06)	.867	2.07 (.27–15.67)	.480	...	...	2.51 (1.05–6.02)	.039*	1.55 (.53–4.50)	.424
ȃȃȃHospitalization^b^	2.00 (.52–7.65)	.311	2.13 (.42–10.81)	.362	...		...		1.52 (.59–3.94)	.385	1.60 (.49–5.22)	.432

*Significant, *P* < .05. Abbreviations: aOR, adjusted odds ratio; CI, confidence interval; OR, odds ratio; Ref, reference; VIDA, Vaccine Impact on Diarrhea in Africa; —, indicates insufficient sample sizes to perform analysis.

Comparison group is female.

Comparison group is none.

## DISCUSSION

Our findings indicate that nonadherence to IMCI guidelines for treatment of diarrhea is prevalent at the study sites in sub-Saharan Africa, manifesting as prescription of antibiotics that are ineffective or unnecessary, or not prescribing antibiotics when they are indicated. Overall, 88% of children with dysentery were prescribed antibiotics, but only 59% of them received the recommended first-line therapy with ciprofloxacin. In addition, 77% of MSD cases who had no apparent indication for therapy were prescribed antibiotics. Unindicated antibiotic prescription was associated with specific clinical features, which varied somewhat from site to site, suggesting that cross-cutting antimicrobial stewardship is needed that is informed by local clinical practices.

Overall, most children with watery diarrhea in The Gambia (58%) and Mali (98%) and 25% in Kenya received an antibiotic that the guidelines deemed unnecessary, corroborating results from other studies indicating the widespread nature of this problem in primary care across low- and middle-income countries (LMICs) [22], with some variation in the frequency and predisposing factors by country [23]. In many instances when antibiotics were prescribed without an apparent indication, a drug was selected that has limited utility for diarrheal pathogens. For example, metronidazole was prescribed for nonbloody diarrhea in approximately 28% of episodes in The Gambia and Kenya and 77% in Mali. Metronidazole is not indicated for pediatric diarrhea unless the child has dysentery that fails to improve with antibiotics directed against shigellosis, and it is not without adverse reactions [24]. Inappropriate prescription of cotrimoxazole was also common at all sites among children with watery diarrhea. The indication for empiric treatment with cotrimoxazole is limited to dysentery, and because resistance is now widespread, it should only be given when susceptibility is known or expected based on local data. Future studies should explore these drivers of decisions on antibiotic prescription to support the development of effective strategies that encourage appropriate, effective, affordable, evidence-based, guideline-driven antibiotics use for management of diarrheal diseases.

Inadequate laboratory capacity, a common problem in Africa, has been suggested as a barrier to effective decision making by clinicians in LMICs regarding the need for antibiotic therapy [25–27]; however, this may not fully explain our findings. Although bacterial culture results could be accessed by clinicians caring for VIDA participants, the results were not available for several days after the patients had been treated and so were rarely used for decision making. Recognizing the limitations of culture to inform therapy for dysentery, IMCI advises that antibiotic treatment be guided by local susceptibility patterns, but numerous studies suggest this is not being done. For example, in 2014, Odhiambo et al [28] reported that clinicians at the leading referral hospitals in proximity to the VIDA sites in Kenya did not utilize the existing laboratory capacity on enteric pathogen susceptibility to guide patient management [29–33]. Clinic-based surveillance for diarrheal pathogens was established in this area of Kenya in 1997. Despite a series of publications from these sites demonstrating high rates of AMR to the antibiotics commonly used to treat dysentery [25–27], only approximately 44% of Kenyan children with dysentery in VIDA were treated with the recommended first-line ciprofloxacin therapy. In Mali, no dysentery cases were prescribed ciprofloxacin, whereas metronidazole was prescribed as first-line therapy for 83% of dysentery cases despite its lack of efficacy for shigellosis. Although emergence of resistance to cotrimoxazole prompted the current WHO recommendation that it only be used when local susceptibility is documented, it was prescribed for 68% of dysentery episodes in Mali. In fact, more than 80% of *Shigella* isolates were resistant to cotrimoxazole during GEMS [20] and VIDA [34]. Prescription of these ineffective drugs for unnecessary indications presents risk without benefit and may, in part, explain the frequent poor treatment outcomes and antibiotic resistance in these countries and other LMICs [27].

Antibiotics were prescribed significantly more often in our study when selected clinical findings were present that do not carry an indication. For example, in Mali and Kenya, antibiotic prescription was 2–4 times more likely when a healthcare provider observed signs of dehydration (dry mouth, excessive thirst, and/or decreased skin turgor). In The Gambia and Kenya, both rural sites, children with cough in the absence of respiratory distress were more likely to receive antibiotics than those without these findings. These observations corroborate those of Rhee et al [22] in Kenya and Ahmad et al [32] in India that cough without IMCI criteria for pneumonia was frequently associated with unnecessary antibiotic prescribing among children with diarrhea.

The main limitation in our current study is that it was not originally designed to evaluate the decision process in antibiotic prescribing, which could have resulted in misclassification of some cases as not warranting antibiotics. Important factors that this study did not explore include the prescribers’ knowledge, attitude, and rationale for their antibiotic prescription decisions or barriers to implementing guidelines such as drug cost or availability.

## Conclusions

The WHO considers AMR to be one of the biggest threats to global health, food security, and development, and underscores the role of antibiotic misuse in accelerating the process [5]. The high prevalence of antibiotic prescriptions that are both unindicated and ineffective underscores the compelling need to explore the factors that drive clinical decision making, the barriers to implementing antimicrobial stewardship in LMICs [35], and the feasibility and effectiveness of interventions that might influence a clinician's decision to prescribe a recommended antibiotic in these settings [36–38]. Research, policy, and implementation must be harnessed to develop robust antibiotic stewardship that includes healthcare worker training in treatment guidelines and the implications of nonadherence, dissemination of local antibiotic susceptibility data in a format that informs treatment decisions, increasing access to affordable first-line antibiotics at health centers while limiting access to antibiotic dispensing by untrained persons, community messaging about prudent antibiotic use, and ongoing oversight.

## Supplementary Data

Supplementary materials are available at *Clinical Infectious Diseases* online. Consisting of data provided by the authors to benefit the reader, the posted materials are not copyedited and are the sole responsibility of the authors, so questions or comments should be addressed to the corresponding author.

## Supplementary Material

ciac980_Supplementary_Data

## Notes

***Acknowledgments.*** This study includes data generated from the site-specific demographic surveillance system (DSS). Therefore, the authors appreciate the contributions and support from the DSS and Ministry of Health from the individual study sites. They appreciate the contributions and support from study staff and leadership at the individual sites including University of Maryland, Baltimore, EMMES, University of Virginia, and Bill & Melinda Gates Foundation (BMGF). They thank the study participants and their families for their voluntary participation.

***Financial support.*** This work was supported by the Bill & Melinda Gates Foundation (grant number OPP1111236/OPP1116751).

***Supplement sponsorship*.** This supplement was sponsored by the Bill & Melinda Gates Foundation. This study is based on research funded in part by grants from the Bill & Melinda Gates Foundation (OPP1111236/OPP1116751).
