# Supplementary material for: Antibiotic-Prescribing Practices for Management of Childhood Diarrhea in 3 Sub-Saharan African Countries: Findings From the Vaccine Impact on Diarrhea in Africa (VIDA) Study, 2015–2018
Source: Clin Infect Dis. 2023 Apr 19;76(Suppl 1):S32–40. doi: 10.1093/cid/ciac980 (PMC10116514; doi:10.1093/cid/ciac980)
Supplement: ciac980_Supplementary_Data [file ciac980_supplementary_data.docx]

**SUPPLEMENTARY APPENDIX**

**Antibiotic prescribing practices for management of childhood diarrhea in three sub-Saharan African countries: Findings from the Vaccine Impact on Diarrhea in Africa (VIDA) study, 2015-2018.**

Alex O. Awuor, Billy Ogwel, Helen Powell, Jennifer R. Verani, Samba O. Sow, M. Jahangir Hossain, John B. Ochieng, Jane Juma, Leslie P. Jamka, Anna Roose, Sanogo Doh, Emily L. Deichsel, Uma Onwuchekwa, Adama Mamby Keita, Martin Antonio, Joquina Chiquita M. Jones, Syed M.A. Zaman, Henry Badji, Irene N. Kasumba, Dilruba Nasrin, James A. Platts-Mills, Eric R. Houpt, David M. Berendes, Ciara E. Sugerman, Marc-Alain Widdowson, Sharon M. Tennant, Eric D. Mintz, Richard Omore, Karen L. Kotloff

**Supplementary Table 1.** Integrated Management of Childhood Illness (IMCI) and other clinical symptoms which are routinely treated with antibiotics

**Supplementary Table 2.** Number and proportion of MSD cases excluded for meeting non-diarrheal criteria for antibiotic prescription stratified by site in VIDA, 2015-2018

**Supplementary Table 1. Integrated Management of Childhood Illness (IMCI) and other clinical symptoms which are routinely treated with antibiotics**

| **IMCI Clinical symptom** | **IMCI Description** | **VIDA questions used to determine if the clinical symptom was present** |
| --- | --- | --- |
| Uncomplicated SAM | WHZ < -3 or  MUAC < 115mm (11.5cm) and  Able to finish a ready-to-use therapeutic food (RUTF) | **WHZ and MUAC:**  Study staff took anthropometric measurements at the time of enrollment including:   - weight in kg (with and without the caretaker for children under 2 months) - mid-upper arm circumference (MUAC) in cm (measured 3 times for accuracy) - height in cm (measured 3 times for accuracy)   The child’s weight-for-height z-score (WHZ) was estimated using the WHO z-score method and the median of the three weight measurements. The median of the three MUAC measurements was estimated.  **RUTF:**  There were no questions at the time of enrollment which captured information about the child’s ability to finish RUTF. |
| Complicated SAM | WHZ < -3 or  MUAC < 115mm (11.5 cm) or  bipedal edema and  medical complication (  any general danger sign or  any other severe classification or pneumonia with chest indrawing) or  not able to finish RUTF (6 months or older) or  breastfeeding problem (less than 6 months) | **WHZ and MUAC:**  Described previously for uncomplicated SAM  **Bipedal edema:**  Presence of bipedal edema was reported by study staff.  **Any general danger sign:**  The caregiver was asked if the child had been unable to drink, experienced convulsing, loss of consciousness, decreased activity or lethargy since the diarrheal illness began. The caregiver was also asked if the child had experienced any vomiting or if they were currently (at the time of enrollment) drinking poorly/not able to drink or lethargic/had loss of consciousness. The child being lethargic or unconscious could also be reported by the study staff.  **Any other severe classification:**  The following severe classification and outlined in this table:   - Severe or very severe pneumonia - Very severe Febrile Disease - Uncomplicated severe Acute Malnutrition   A child with at least two of the following was considered to have severe dehydration:   - Caregiver reported a child drinking much less than usual or being unable to drink - Study staff recorded the child was lethargic or unconscious - Study staff recorded the eyes were sunken (confirmed with the caregiver that the eyes were more sunken than usual) - Study staff recorded a very slow (> 2 seconds) skin pinch return rate   Neither mastoiditis or severe anemia were recorded but could be noted by study staff as the diagnosis at the time of hospital discharge.  **Pneumonia with chest indrawing:**  Pneumonia is outlined in this table and those who met this definition due to chest indrawing, as opposed to fast breathing, are considered here.  **RUTF/Breastfeeding:**  There were no questions at the time of enrollment which captured information about the child’s ability to finish RUTF or difficulties with breastfeeding. |
| Pneumonia | Cough or difficulty breathing + chest indrawing or fast breathing  Fast breathing is defined as 50 or more breaths per minute for a child 2 months up to 12 months or 40 or more breaths per minute for a child 12 months up to 5 years. | **Cough/difficulty breathing:**  A cough or difficulty breathing, since the diarrheal illness began, could be reported by the caregiver at the time of enrollment. A cough could also be recorded by study staff as a diagnosis at hospital discharge.  **Chest indrawing:**  Chest indrawing was recorded by study staff  **Fast breathing:**  Respiratory rate per minute was recorded twice by study staff at enrollment and the average of these two measurements was used in conjunction wit the child’s calculated age (based on provided DOB) to determine if fast breathing was present.  Pneumonia can also be indicated as a diagnosis at hospital discharge and would be recorded by study staff. |
| Severe or very severe pneumonia | Cough or difficulty breathing +  a general danger sign or  stridor in a calm child | **Cough/difficulty breathing:**  Described previously for pneumonia  **Any general danger sign:**  Described previously for complicated SAM  **Stridor in a calm child:**  There were no questions asked at the time of enrollment which captured information about stridor. |
| Dysentery | Blood in the stool | Blood in the stool can be reported by the caregiver or witnessed by the interviewer or study staff at the time of enrollment. |
| Acute Ear Infection | Ear pain or  pus is seen draining from the ear and discharge is reported for less than 14 days | There were no direct questions asked about ear pain or pus/discharge from the ear. However, at the time of hospital discharge a diagnosis could be recorded in a free text field. Those with indications of ear pain will be assumed to have an acute ear infection. |
| Very severe febrile disease | Regardless of malaria risk:  Child fells hot or has a temperature of 37.5$℃$ or above +  any general danger sign or  a stiff neck | **Fever:**  A fever, since the diarrheal illness began, could be reported by the caregiver. Study staff also measured to child’s axilliary temperature at the time of enrollment.  **Any general danger sign:**  Described previously for complicated SAM  **Stiff neck:**  There were no questions asked at the time of enrollment which captured information about stiff neck. |
| **Other clinical symptoms** | | |
| Bacterial infection | | An invasive bacterial infection can be indicated as a diagnosis at hospital discharge and would be recorded by study staff. |
| Tonsillitis | | Tonsillitis can be indicated in the free text field as a diagnosis at hospital discharge and would be recorded by study staff. |
| Pharyngitis | | Pharyngitis can be indicated in the free text field as a diagnosis at hospital discharge and would be recorded by study staff. |
| Meningitis | | Meningitis can be indicated as a diagnosis at hospital discharge and would be recorded by study staff. |
| Septicemia | | Septicemia can be indicated in the free text field as a diagnosis at hospital discharge and would be recorded by study staff. |
| Impetigo | | Impetigo can be indicated in the free text field as a diagnosis at hospital discharge and would be recorded by study staff. |

Abbreviations: RUTF, ready to use therapeutic food; IMCI, Integrated Management of Childhood Illness guidelines from the World Health Organization; WHZ, weight for height z score; MUAC, mid-upper arm circumference; SAM, severe acute malnutrition.

**Supplementary Table 2. Number and proportion of MSD cases excluded for meeting non-diarrheal criteria for antibiotic prescription stratified by site in VIDA, 2015-2018**

| **Exclusion criteria** | | **The Gambia (N=809)** | **Mali**  **(N=468)** | **Kenya (N=1,066)** |
| --- | --- | --- | --- | --- |
|  |  | n (%) | n (%) | n (%) |
| **Clinician Diagnosis** | Other invasive bacterial infection | 11 (1) | 2 (0) | 123 (12) |
|  | Pneumonia | 132 (16) | 42 (9) | 73 (7) |
|  | Meningitis | 1 (0) | 0 (0) | 0 (0) |
|  | Otitis Media | 3 (0) | 3 (1) | 1 (0) |
|  | Pharyngitis | 0 (0) | 12 (3) | 0 (0) |
|  | Skin infection | 7 (1) | 0 (0) | 4 (0) |
|  | Sepsis | 0 (0) | 0 (0) | 3 (0) |
|  | Impetigo | 0 (0) | 0 (0) | 2 (0) |
| **IMCI Case Definitions** | Uncomplicated SAM | 33 (4) | 41 (9) | 6 (1) |
|  | Pneumonia | 145 (18) | 39 (8) | 135 (13) |
|  | Severe pneumonia | 331 (41) | 176 (38) | 570 (53) |
|  | SAM with complications | 147 (18) | 40 (9) | 45 (4) |
|  | Very severe febrile diseases | 257 (32) | 177 (38) | 323 (30) |
